# Supplementary material for: Cdk9 regulates a promoter-proximal checkpoint to modulate RNA polymerase II elongation rate in fission yeast
Source: Nat Commun. 2018 Feb 7;9:543. doi: 10.1038/s41467-018-03006-4 (PMC5803247; doi:10.1038/s41467-018-03006-4)
Supplement: Supplementary file 1 — Supplementary Information [file 41467_2018_3006_MOESM1_ESM.pdf]

Cdk9 regulates a promoter-proximal checkpoint to modulate RNA Polymerase II elongation rate in fission yeast

Booth *et. al.*

SUPPLEMENTARY INFORMATION

Supplementary Figure 1

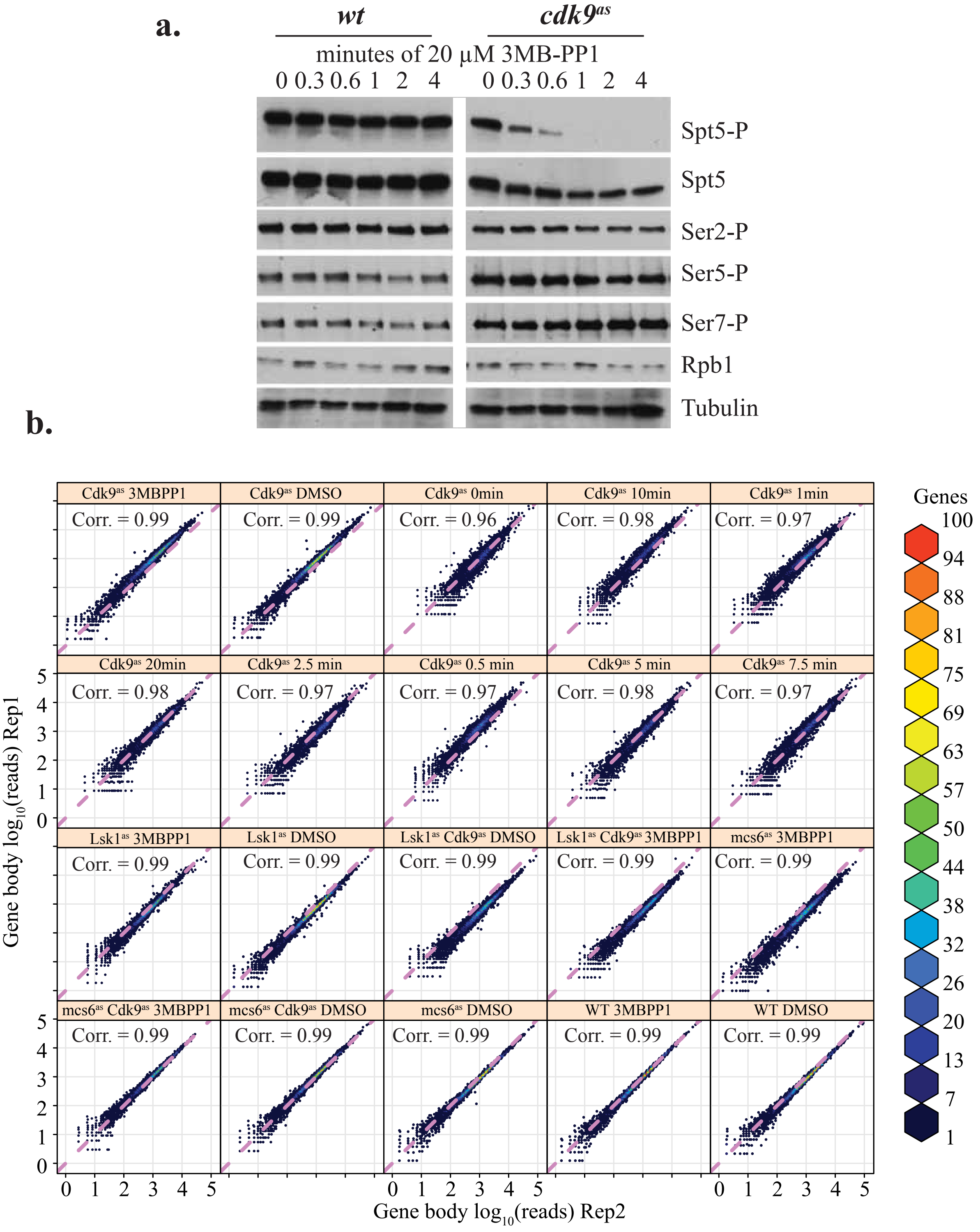

Supplementary Figure 1 | *Cdk9* inhibition primarily affects *Spt5*-P within a short timeframe and PRO-seq experiments are reproducible. a. Western blot analysis of phosphorylated

residues within the elongation complex in *wt* and *cdk9*^{as} strains after increasing durations of treatment with 20 μM 3-MB-PP1. From top to bottom, antibodies were raised against, *Spt5*-P, total *Spt5*, Ser2-P, Ser5-P, Ser7-P, *Rpb1*, or *Tubulin* (loading control). **b.** Scatter plots displaying a correlation (Spearman's rho) between biological replicate PRO-seq data for each sample. Data points show spike-in normalized read counts ($\log_{10}$) within the gene body (TSS + 200 bp to annotated CPS, where "+" indicates downstream of TSS) of all filtered genes. Since minimal variation is expected between replicates, accurate spike in-based normalization will produce scatter that is approximately centered on the purple diagonal line ($x = y$).

Supplementary Figure 2

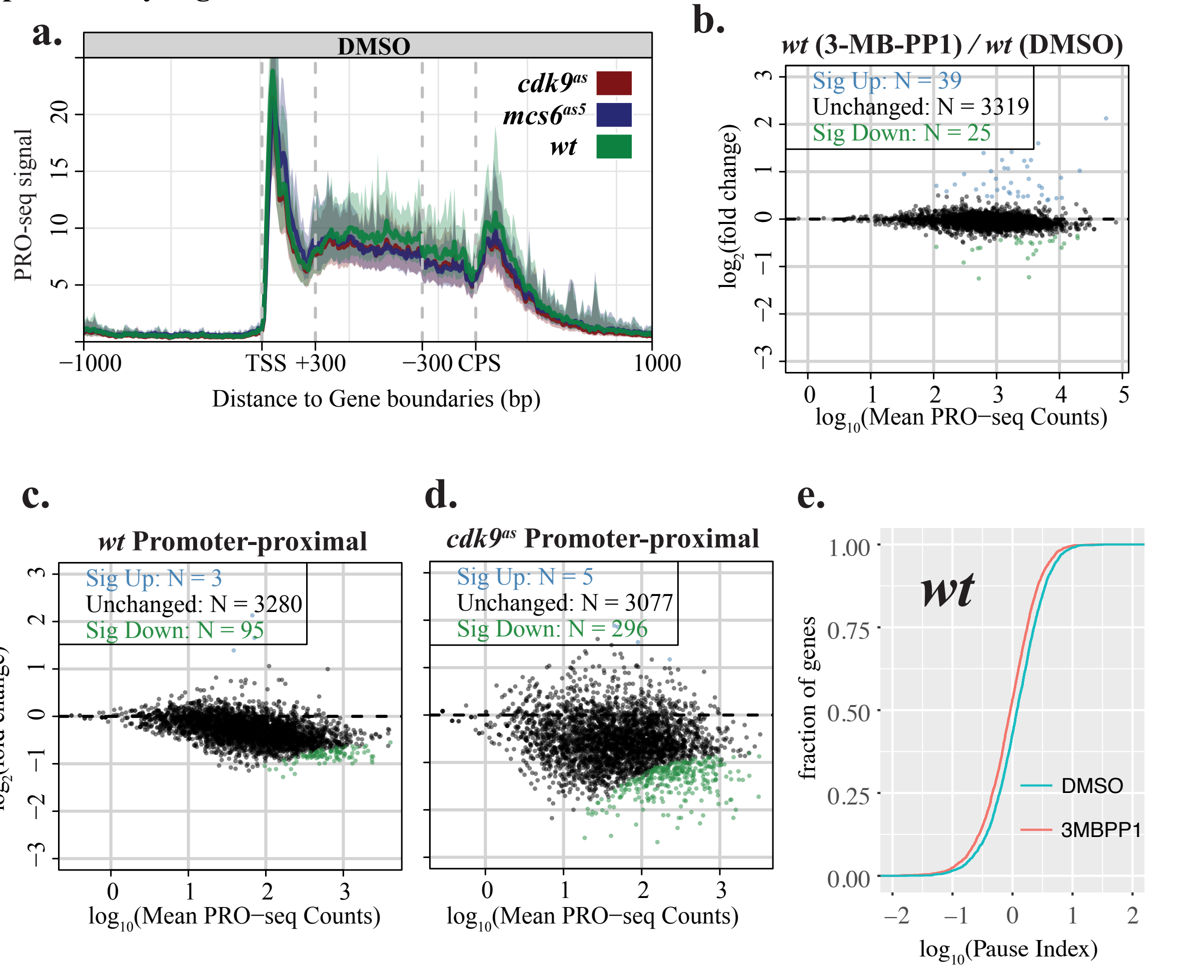

Supplementary Figure 2 | AS strains provide a highly controlled system for studying kinase impact on transcription. **a.** Composite profiles of combined replicate data for samples of untreated wild type, $mcs6^{as5}$, and $cdk9^{as}$ strains across filtered genes separated from nearest same strand neighbors by at least 1 kb on both sides (n = 939). Composite profiles represent the median subsampled value from combining two biological replicates within each bin. Shaded regions correspond to the 12.5% and 87.5% quantiles. To scale genes to a common length, the middle gene body region of each gene was scaled to 60 bins, while the regions -1000 bp to +300 bp relative to the TSS, and -300 bp to +1000 bp relative to the CPS, are unscaled 10 bp windows ("+" and "-" indicate downstream and upstream, respectively). **b.** MA plot displaying the $\log_2$ fold change between gene body read counts from treated and untreated *wt* cells, as calculated using the DESeq2 package[1]. **c & d.** MA plot displaying the $\log_2$ fold change between treated and untreated promoter proximal (TSS to +100 bp) read counts for wild type (**c.**), or $cdk9^{as}$ (**d.**). Genes that show significantly increased or decreased PRO-seq signal (adjusted p <0.01; DESeq2: Wald test, Benjamini and Hochberg's correction) are shown in blue and green, respectively. For all MA plots, spike-in normalization was used when calculating $\log_2$ fold changes between samples. **e.** Cumulative density functions for pausing index ($\log_{10}$) of all filtered genes in treated (red) and untreated (blue) samples for the wild type strain.

Supplementary Figure 3

a.

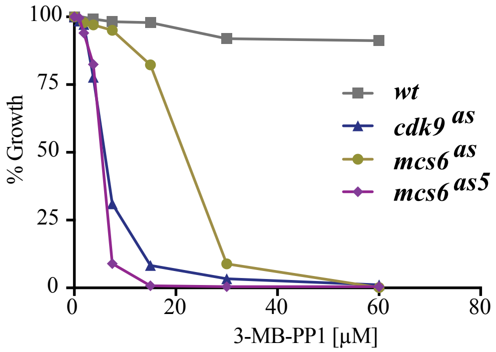

b.

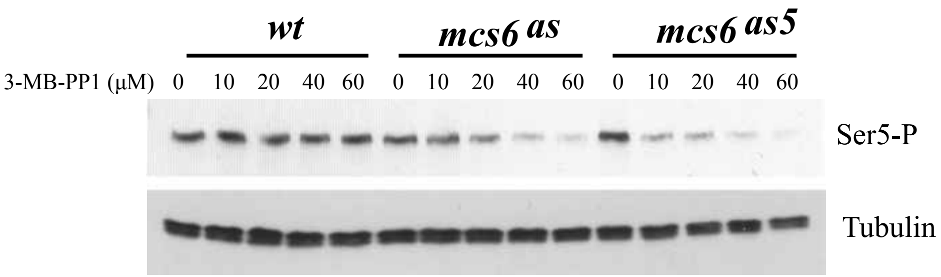

c.

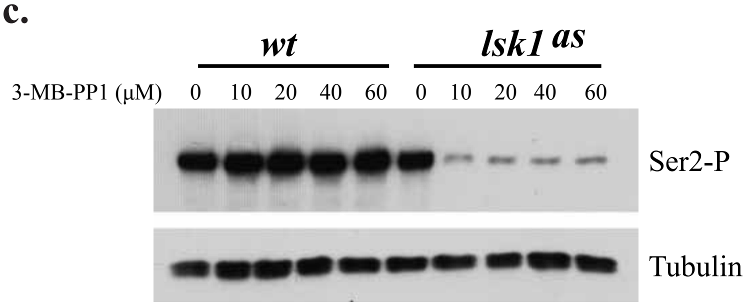

Supplementary Figure 3 | Characterization of AS strains and the effects of 3-MB-PP1 concentration. a. The indicated strains were grown in 96-well plates in the presence of increasing amounts of 3-MB-PP1 and their growth quantified by OD_{600}. The mutant alleles and their associated mutations are: *mcs6*^{as}, L87G; *mcs6*^{as5}, N84T/L87G; *cdk9*^{as}, T120G. **b.** Western blot analysis of Ser5-P relative to tubulin (loading control) in *wt*, *mcs6*^{as}, and *mcs6*^{as5} strains after 1 hr treatment at 30 °C with the specified concentration of 3-MB-PP1. **c.** Western blot analysis of Ser2-P relative to *tubulin* in *wt*, and *lsk1*^{as} strains after 1 hr of treatment at 30 °C with the specified concentration of 3-MB-PP1.

Supplementary Figure 4

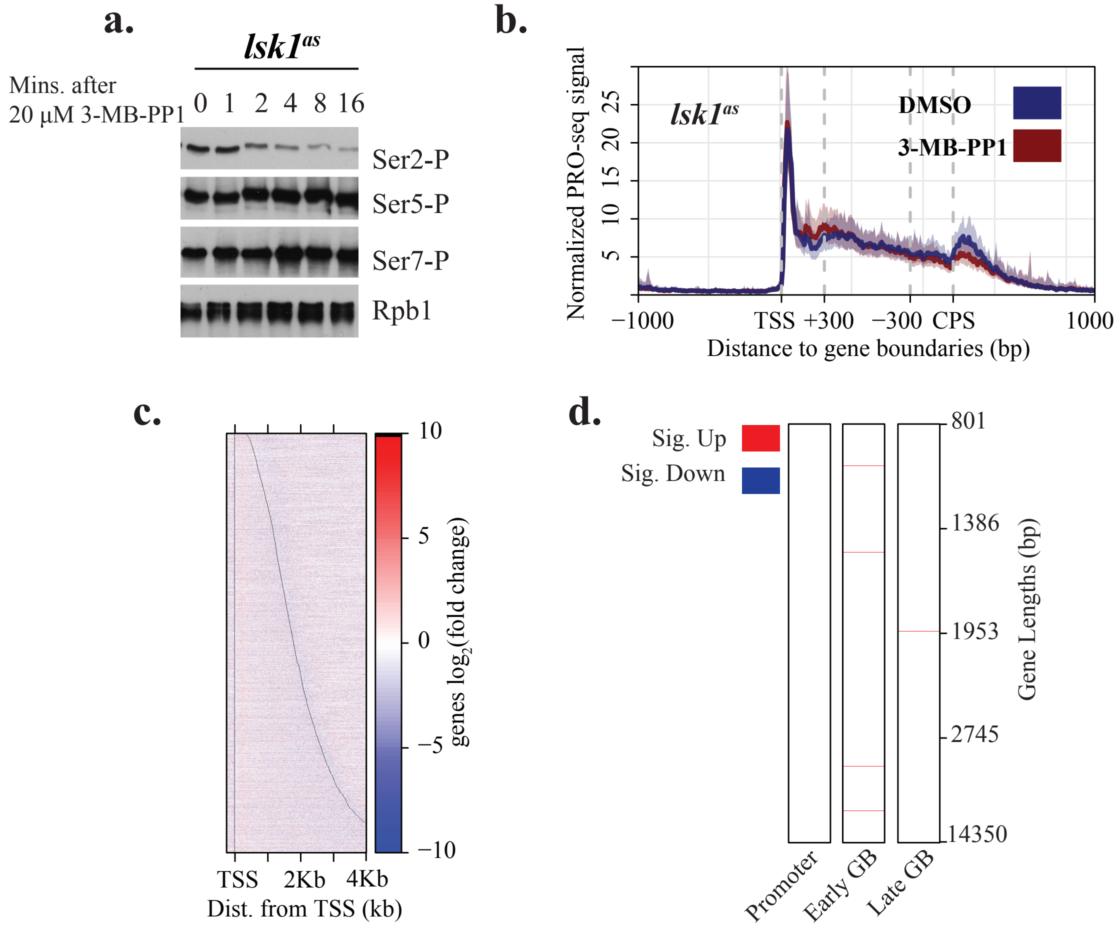

Supplementary Figure 4 | Treatment of *lsk1^as^* with 3-MB-PP1 has minimal impact on transcription within five minutes. a. Western blot analysis of phosphorylated *Pol II* CTD residues (Ser2-P, Ser-5-P, Ser7-P) in relation to total *Rpb1* signal. Levels were measured over a time course of *lsk1^{as}* treated with 20 µM 3-MB-PP1. **b.** Composite PRO-seq profiles for treated (5 min 10 µM 3-MB-PP1) and untreated (5 min DMSO) *lsk1^{as}* displaying median subsampled signal across filtered genes separated from nearest same strand neighbors by at least 1 kb on both sides (n = 939). Shaded regions correspond to the 12.5% and 87.5% quantiles. The middle gene body region of each gene was scaled to 60 bins, while the regions, -1000 bp to +300 bp, relative to the TSS, and -300 bp to +1000 bp relative to the CPS, are unscaled 10 bp windows. **c.** Heat map depicting the $\log_2$ fold change in normalized PRO-seq signal (treated/untreated) in *lsk1^{as}* within 10 bp bins from -250 bp to +4000 bp relative to the TSS. Genes are sorted by increasing length, with black lines representing observed TSS and annotated CPS. Data shown in b. & c. represent combined data from two biological replicates. **d.** Heat maps depicting whether each gene exhibits a significant fold change (adjusted $p < 0.01$; treated/untreated; DESeq2: Wald test, Benjamini and Hochberg's correction) in promoter, early, or late gene body regions (defined in Figure 2). Genes were required to be longer than 800 nt and are sorted from top to bottom by increasing gene length (n = 3003). Gene length quartiles are shown with tick marks on the right. Significant increases and decreases in each region are shown as red and blue, respectively.

Supplementary Figure 5

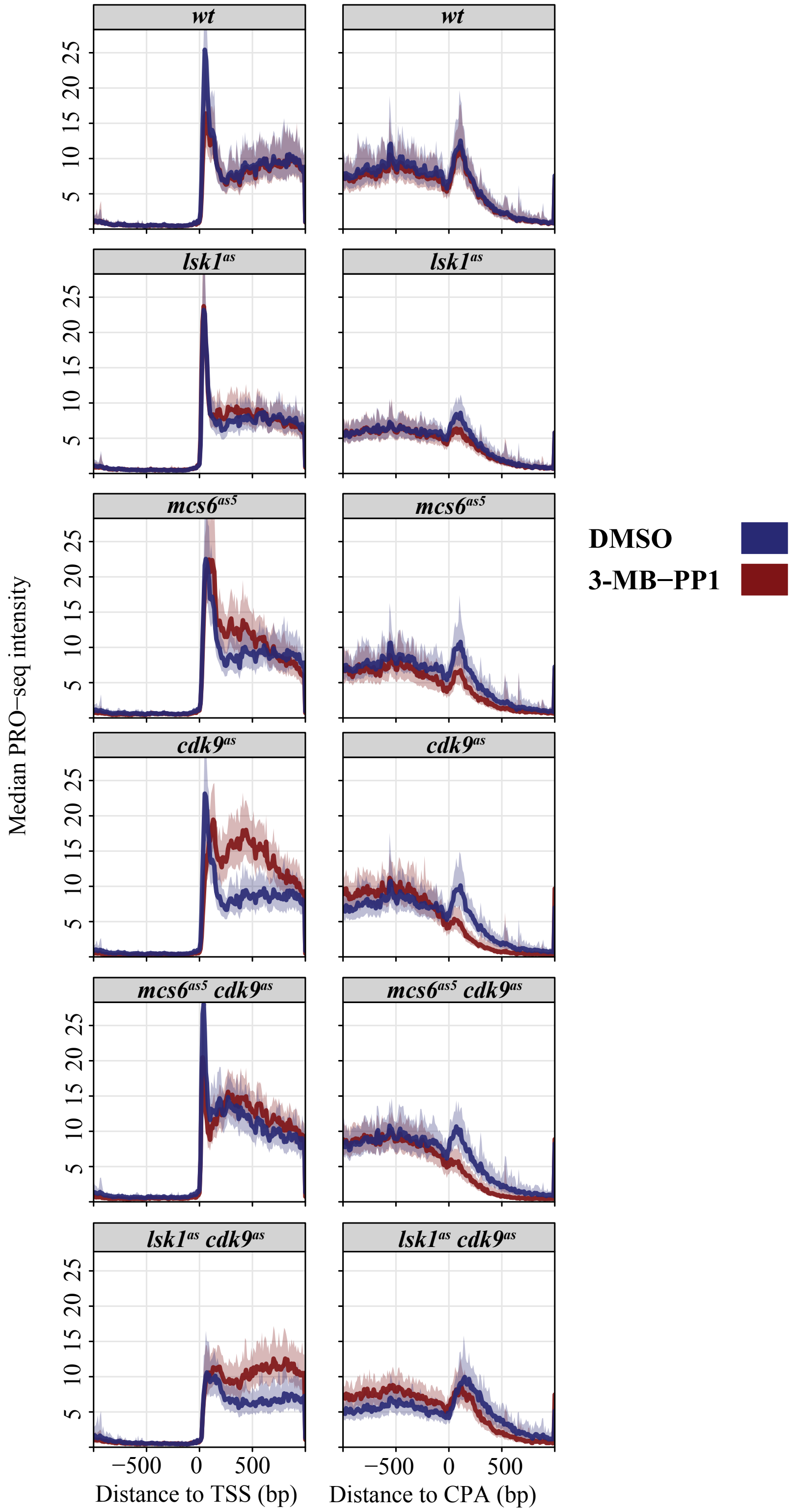

Supplementary Figure 5 | Global effects of kinase inhibition revealed through composite profiles centered on TSS and CPS. Each panel displays centered composite PRO-seq profiles of treated (5 min 10 μM 3-MB-PP1) and untreated (5 min DMSO) samples from each strain. Dark middle lines reflect the median sub-sampled PRO-seq signal within 10 bp windows from -1 kb to +1 kb from observed TSS (left) or CPS (right) for genes separated from nearest same strand neighbors by at least 1 kb on both sides (n = 939). Shaded regions correspond to the 12.5% and 87.5% quantiles. For the combined mutants, composite untreated PRO-seq profiles appear to deviate from those of the *wt* strain, indicating a possible synthetic effect of the mutations on transcription. Therefore, we were limited in our ability to interpret the results from these strains. With the exception of 3-MB-PP1-treated *mcs6*^{as5} (see methods), all profiles represent combined data from two biological replicates.

Supplementary Figure 6

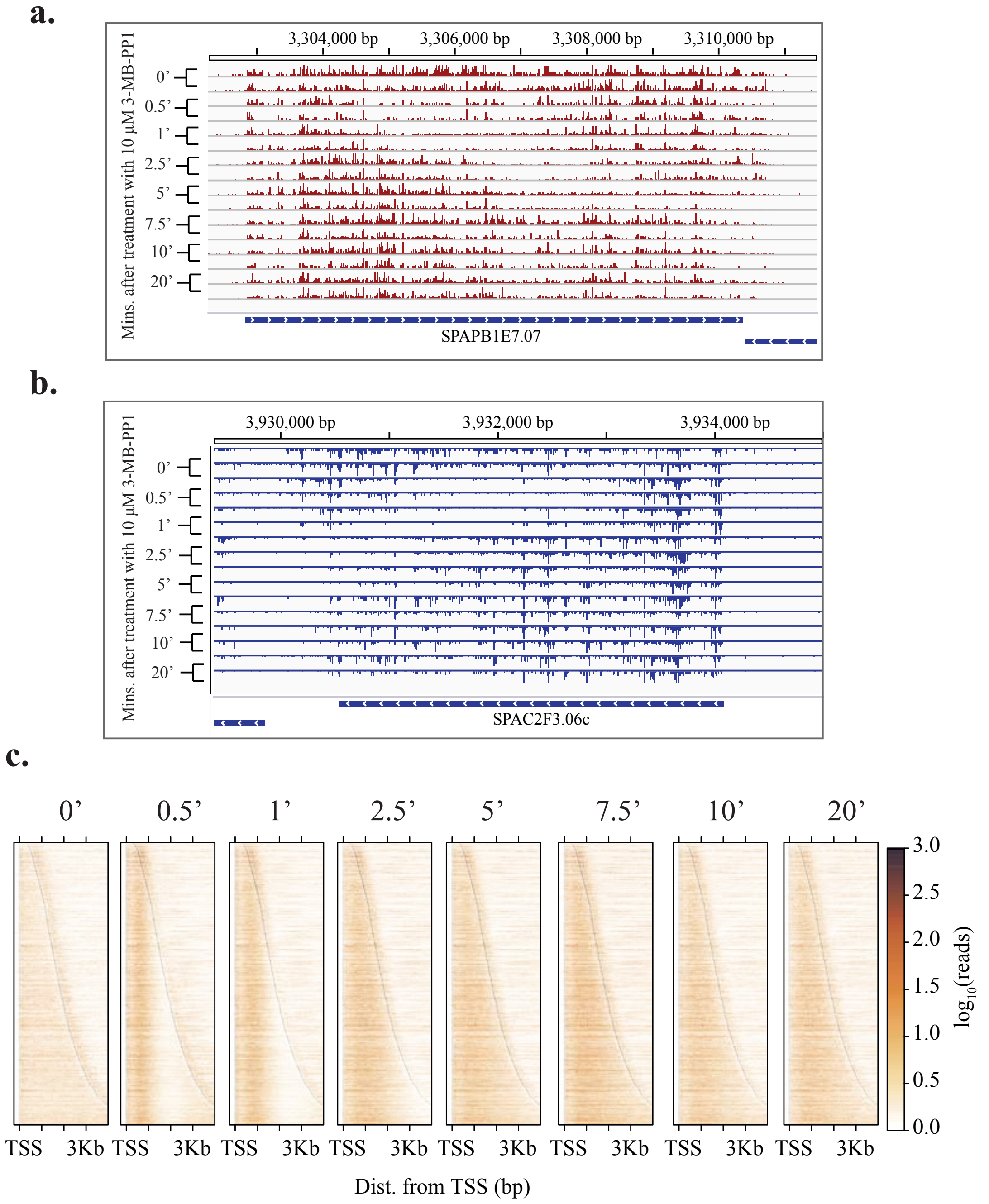

Supplementary Figure 6 | Reproducible time-dependent impact of *Cdk9* inhibition on transcription. a & b. Browser track images from the *SPAPB1E7.07* (**a.**) and *SPAC2F3.06c* (**b.**) loci. Tracks represent normalized read counts from separate biological replicates of *cdk9^{as}* on the plus or minus strands, respectively, treated with 10 μM 3-MB-PP1 for increasing amounts of time. **c.** Raw heatmaps of PRO-seq signal for strains before and after treatment. Heatmap signal intensity reflects normalized signal ($\log_{10}$) within 10 bp bins from -250 to +4000 bp relative to the TSS for data from combined replicates for increasing treatment durations in *cdk9^{as}*, from left

to right. Within each panel, genes are sorted by increasing length from top to bottom, with dark lines showing boundaries for each gene. All samples were treated with 10 μM 3-MB-PP1 for the specified times. The zero-minute treatment was treated with DMSO for 20 min. All heatmaps reflect combined data from two biological replicates.

Supplementary Figure 7

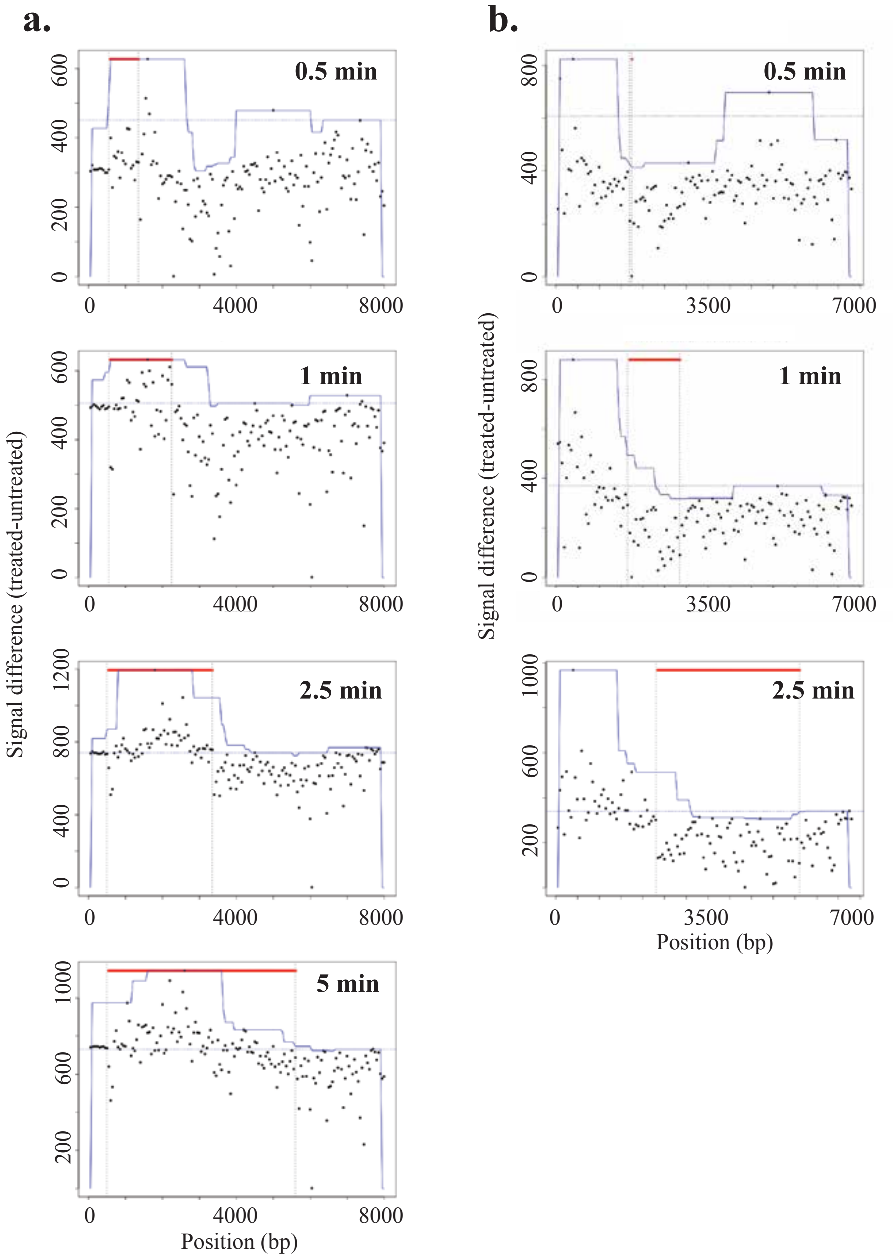

Supplementary Figure 7 | Examples of difference maps used for calling waves at each time point. **a.** Advancing wave location estimates based on difference in PRO-seq signal (treated – untreated) measured within 50 bp windows over the *SPAPB1E7.07* locus at each time point (0.5 min, 1 min, 2.5 min, and 5 min). **b.** Estimates of region between advancing and clearing waves based on difference in PRO-seq signal (treated – untreated) measured within 50 bp windows over the *SPAC926.09c* locus at each time point (0.5 min, 1 min, 2.5 min). For each plot, the y-axis displays the difference within each 50 bp window plus a constant (dotted horizontal line), which makes the minimum value equal to one. The red points indicate windows identified as being within the advancing wave (a) or between waves (b). The blue trace reflects the local maximum within a moving 1 kb window.

Supplementary Figure 8

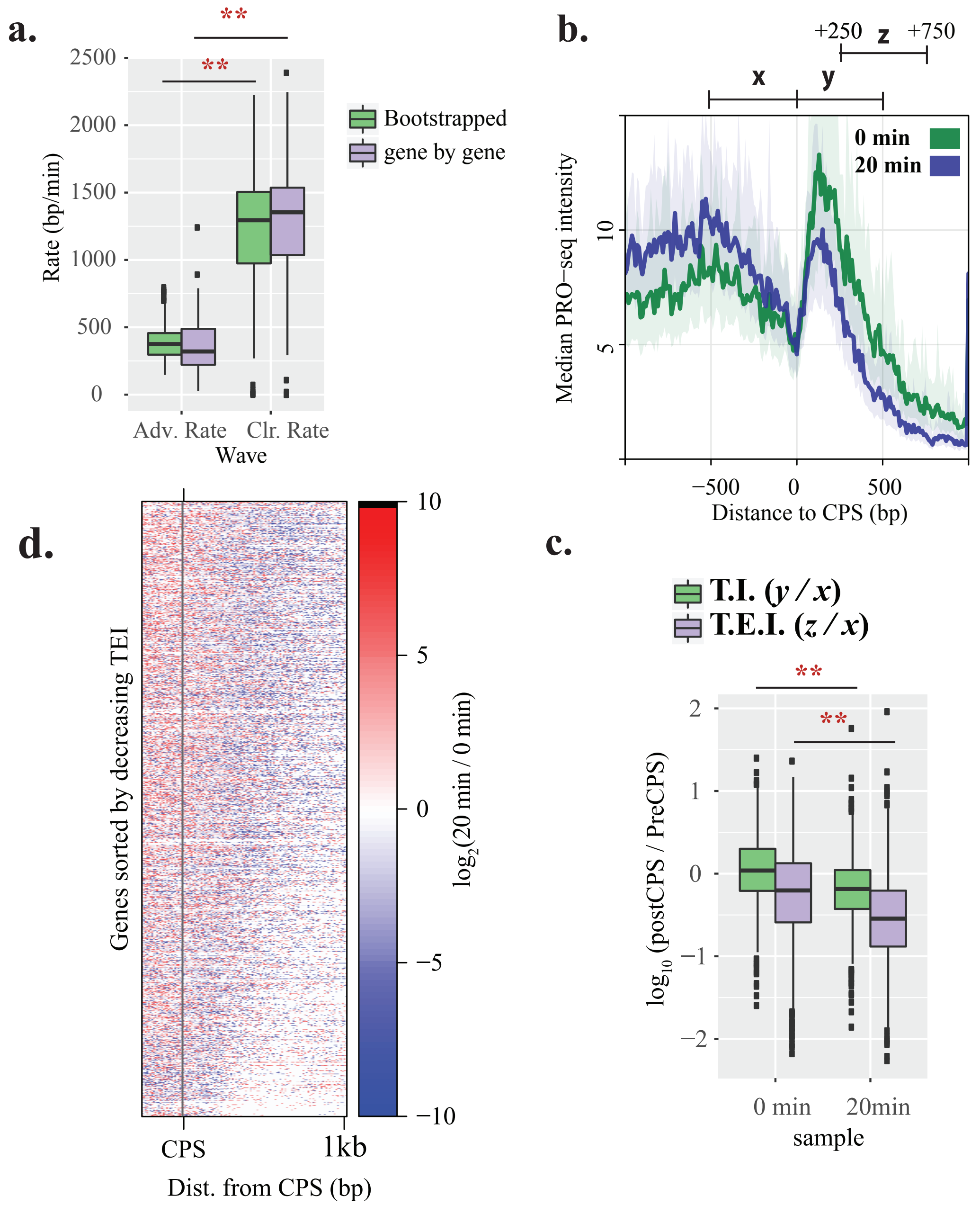

Supplementary Figure 8 | Decreased Pol II transcription rates globally reduce post-CPS elongation upon continued *Cdk9* inhibition. a. Box plots show distributions of rate estimates for the advancing wave (left) and clearing wave (right). Significant differences were observed between advancing and clearing rates using both bootstrapping ($p < 2.2\text{e-}16$, Student's t-test) and gene-by-gene ($p = 9.619\text{e-}08$, Student's t-test) approaches. For each boxplot, the centerline represents the median, the bounds of the box represent the 1st and 3rd quartiles, and the whiskers extend 1.5 times the interquartile range. **b.** CPS centered composite PRO-seq profiles comparing *cdk9^{as}*, either untreated (green) or after 20 min of treatment with 10 µM 3-MB-PP1 (blue). Dark middle lines reflect the median subsampled PRO-seq signal within 10 bp windows from -1 kb to

+1 kb of the CPS for genes separated from nearest same strand neighbors by at least 1 kb on both sides (n = 939). Shaded regions correspond to the 12.5% and 87.5% quantiles. **c.** Distributions of termination index (T.I.; green) and termination elongation index (T.E.I., purple) for all genes used in (b) before (left) and after 20 min of *Cdk9* inhibition (right). T.I. and T.E.I. are metrics that capture elongation beyond the CPS, relative to signal in the late gene body. All 500 bp regions used for calculating T.I. (y / x) and T.E.I. (z / x) ratios are depicted above the plot in (b). **d.** Heatmap of $\log_2$ fold change (20 min/ 0 min) within 10 bp windows from -250 to +1000 bp relative to CPS for genes used in (b) and (c). Genes are sorted by decreasing T.E.I. from top to bottom as calculated from untreated *cdk9*^{as}. Data from combining two replicates for each treatment was used for all plots and calculations.

Supplementary Table 1 | Yeast strains

Strain	Allias	Genotype	Reference
JS78	*wt*	*leu1-32 ura4-D18 his3-D1 ade6-M210 h^+*	Saiz & Fisher 2002
LV7	*$cdk9^{as}$*	*$cdk9^{T120G}$::kanMX6 leu1-32 ura4-D18 his3-D1 ade6-M210 h^+*	Viladevall et al. 2009
MS340	*$mcs6^{as5}$*	*$mcs6^{N84T -L87G}$::kanMX6 leu1-32 ura4-D18 his3-D1 ade6-M210 h^+*	This study
CS118	*$lsk1^{as}$*	*$lsk1^{F353G}$::kanMX6 leu1-32 ura4-D18 his3-D1 ade6-M216 h^-*	Viladevall et al. 2009
PP20	*$cdk9^{as}$ $lsk1^{as}$*	*$cdk9^{T120G}$::hphMX6 $lsk1^{F353G}$::kanMX6 leu1-32 ura4-D18 his3-D1 ade6-M210 h^+*	This study
PP23	*$cdk9^{as}$ $mcs6^{as5}$*	*$cdk9^{T120G}$::hphMX6 $mcs6^{N84T-L87G}$::kanMX6 leu1-32 ura4-D18 his3-D1 ade6-M210 h^-*	This study
S. cereivisae	Spike-in	W303-1a	Booth et al. 2016

Supplementary Table 2 | PRO-seq sample information

Extended Data Table 2: PRO-seq sample information

sample	spike-in ribosomal	ribosomal	spike-in	genome	total	Norm. Factor	batch
WT_DMSOr1	10,174,782	12,285,660	46,236	6,157,670	30,465,302	0.46236	1
WT_DMSOr2	12,204,603	15,627,984	54,480	8,401,775	38,708,110	0.5448	1
WT_3MBPP1_1	12,326,132	15,585,548	80,667	10,653,445	41,242,195	0.80667	1
WT_3MBPP1_2	10,582,750	13,617,874	62,899	8,593,076	35,067,313	0.62899	1
mcs6_DMSO1	12,257,633	15,672,640	80,616	10,205,273	41,454,045	0.80616	1
mcs6_DMSO2	11,707,207	14,554,011	56,817	8,051,885	36,735,682	0.56817	1
mcs6_3MBPP1_1*	11,430,584	13,874,952	101,502	6,331,691	37,568,528	1.01502	1
mcs6_3MBPP1_2	12,052,555	14,402,612	72,480	8,466,890	37,221,280	0.7248	1
CDK9_DMSO1	12,389,079	14,034,425	54,917	6,367,375	34,927,058	0.54917	1
CDK9_DMSO2	12,778,993	16,323,383	66,519	9,358,197	41,353,473	0.66519	1
CDK9_3MBPP1_1	11,143,318	13,228,901	60,777	7,827,810	34,514,302	0.60777	1
CDK9_3MBPP1_2	12,517,108	16,637,590	88,178	12,311,216	45,190,617	0.88178	1
Isk1as_DMSOr1	8,137,437	9,062,002	73,907	6,843,886	29,144,786	0.73907	2
Isk1as_DMSOr2	10,483,655	11,431,617	67,401	8,289,801	35,769,690	0.67401	2
Isk1as_3MBPP1r1	6,935,506	7,256,537	54,000	5,481,567	23,207,739	0.54	2
Isk1as_3MBPP1r2	3,080,010	2,899,634	17,547	2,063,159	12,330,930	0.17547	2
Mcs6as_CDK9as_DMSOr1	7,733,612	8,014,461	37,501	5,750,563	26,364,431	0.37501	2
Mcs6as_CDK9as_DMSOr2	8,149,246	8,775,629	41,878	5,992,027	26,861,563	0.41878	2
Mcs6as_CDK9as_3MBPP1r1	7,206,380	7,247,941	33,500	4,536,765	23,488,806	0.335	2
Mcs6as_CDK9as_3MBPP1r2	6,378,728	6,817,084	36,155	4,582,814	20,834,265	0.36155	2
Cdk9as_0min_DMSO_rep1	941,848	1,294,433	8,443	1,240,837	6,521,490	0.08443	3
Cdk9as_0min_DMSO_rep2	1,065,358	1,504,048	13,568	2,005,311	10,353,030	0.13568	3
Cdk9as_0.5min_10uM_3MBPP1_rep1	3,205,493	3,805,818	24,773	3,807,026	15,454,733	0.24773	3
Cdk9as_0.5min_10uM_3MBPP1_rep2	1,139,637	1,662,171	16,380	1,738,590	7,779,488	0.1638	3
Cdk9as_1min_10uM_3MBPP1_rep1	1,699,913	2,207,482	16,573	2,128,619	9,584,199	0.16573	3
Cdk9as_1min_10uM_3MBPP1_rep2	2,130,628	3,151,162	30,364	3,413,440	12,760,335	0.30364	3
Cdk9as_2.5min_10uM_3MBPP1_rep1	1,753,947	2,386,420	14,135	1,973,114	8,645,482	0.14135	3
Cdk9as_2.5min_10uM_3MBPP1_rep2	2,557,168	3,377,095	23,249	3,621,442	14,120,294	0.23249	3
Cdk9as_5min_10uM_3MBPP1_rep1	2,394,714	3,307,974	20,338	2,812,736	15,961,497	0.20338	3
Cdk9as_5min_10uM_3MBPP1_rep2	2,504,115	2,672,961	26,141	3,742,474	17,870,169	0.26141	3
Cdk9as_7.5min_10uM_3MBPP1_rep1	1,998,776	2,768,515	15,073	2,612,802	16,268,203	0.15073	3
Cdk9as_7.5min_10uM_3MBPP1_rep2	2,662,662	3,131,133	33,314	5,316,423	21,503,832	0.33314	3
Cdk9as_10min_10uM_3MBPP1_rep1	2,228,285	2,681,515	17,655	3,070,909	15,621,102	0.17655	3
Cdk9as_10min_10uM_3MBPP1_rep2	1,000,575	1,441,581	14,946	1,865,992	9,003,041	0.14946	3
Cdk9as_20min_10uM_3MBPP1_rep1	1,059,830	1,275,738	11,469	1,802,836	8,859,279	0.11469	3
Cdk9as_20min_10uM_3MBPP1_rep2	2,100,685	2,635,181	22,006	3,327,234	15,608,295	0.22006	3
Lsk1as_CDK9as_DMSOr1	1,929,149	2,510,779	32,183	2,953,437	10,834,859	0.32183	3
Lsk1as_CDK9as_DMSOr2	2,795,832	3,489,519	34,863	4,400,322	16,293,462	0.34863	3
Lsk1as_CDK9as_3MBPP1r1	1,566,015	2,350,911	24,970	2,915,731	11,162,211	0.2497	3
Lsk1as_CDK9as_3MBPP1r2	2,789,279	3,492,754	30,235	4,278,326	18,766,044	0.30235	3

* Sample omitted from analysis

Supplementary Figure 9

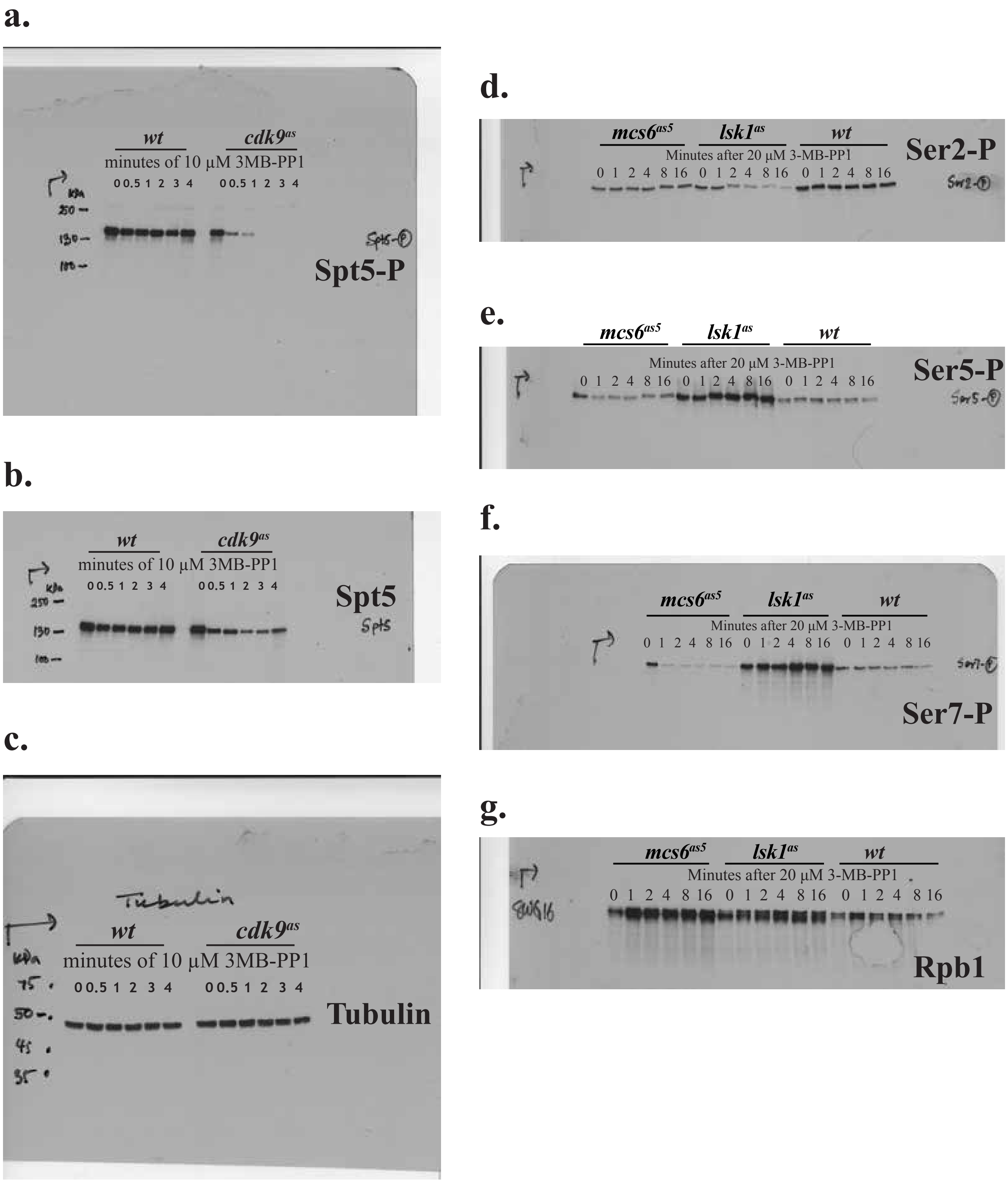

Supplementary Figure 9 | Western blot raw images. a-c. Raw scans of separate western blots used in figure 1a. Total protein extracts were prepared from *wt* and *cdk9*^{as} strains over time-course of treatment with 10 µM 3-MB-PP1. **a.** Western blot using antibody against phosphorylated *Spt5*. **b.** Western blot using antibody against total *Spt5*. **c.** Western blot using antibody against *tubulin*. **d-g.** Raw scans of separate western blots used in figure 2a and Supplementary Figure 4a. Total protein extracts were prepared from *mcs6*^{as5}, *lsk1*^{as}, and *wt*

strains over time-course of treatment with 20 μM 3-MB-PP1. **d-f.** Western blot using antibody against phosphorylated Ser2 (**d**), Ser5 (**e**), or Ser7 (**f**) residues within the CTD of *Rpb1*. **g.** Western blot using antibody against *Rpb1* (8WG16).

Supplementary Figure 10

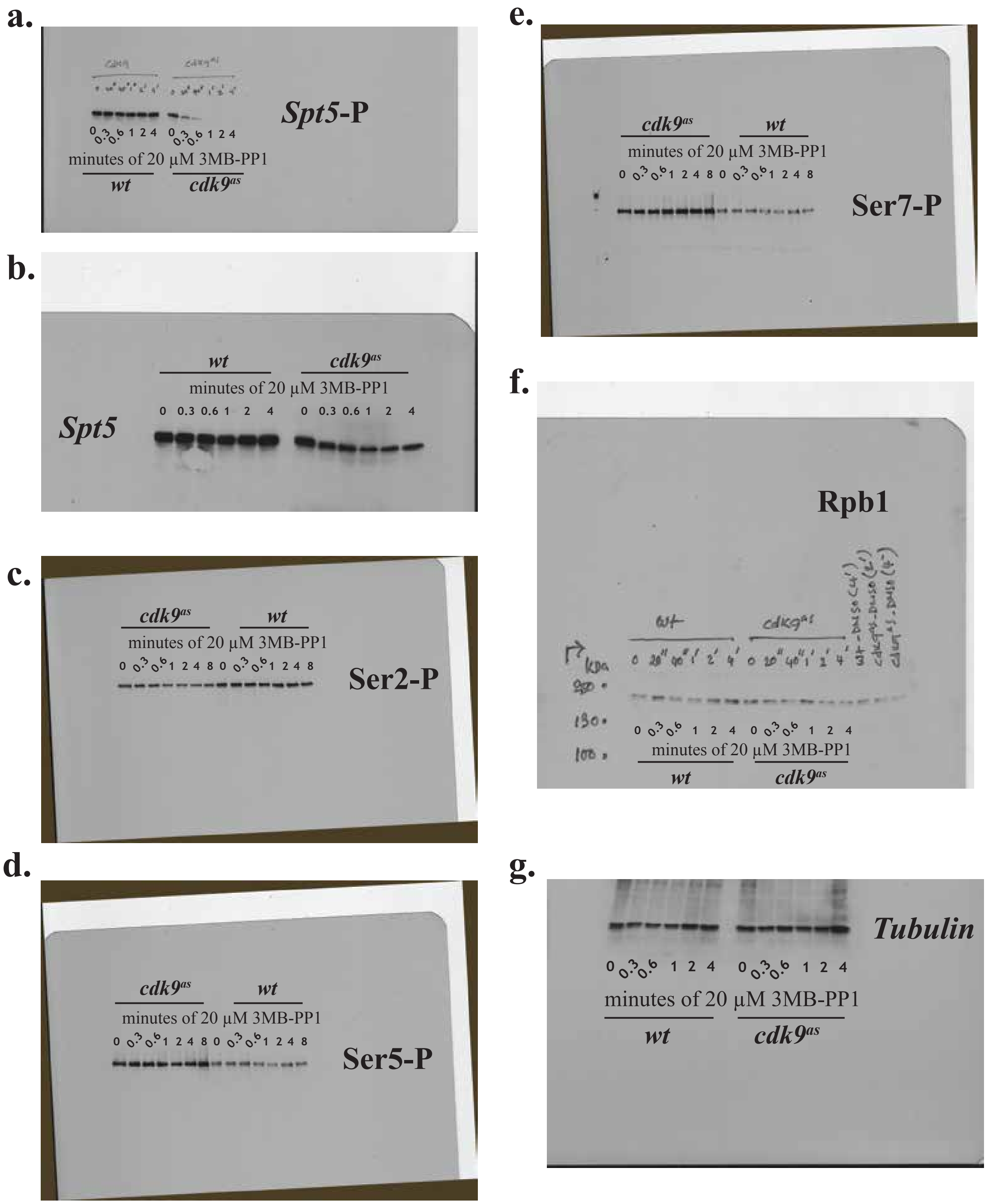

Supplementary Figure 10 | Western blot raw images. Raw scans of separate western blots used in Supplementary Figure 1a. Total protein extracts were prepared from *wt* and $cdk9^{as}$ strains over time-course of treatment with 20 µM 3-MB-PP1. **a.** Western blot using antibody against phosphorylated *Spt5*. **b.** Western blot using antibody against total *Spt5*. **c-e.** Western blot using

antibody against phosphorylated Ser2 (**c.**), Ser2 (**d.**), or Ser7 (**e.**) residues within the CTD of *Rpb1*. **f.** Western blot using antibody against *Rpb1*. On the right of panel f, three additional samples were assayed as follows from left to right, *wt* treated with DMSO for 4 minutes, $cdk9^{as}$ treated with DMSO for 2 minutes, or 4 minutes. **g.** Western blot using antibody against *tubulin*. All gels were run using protein extracts from the same experiments, however, an additional time point (8-minute treatment with 20 μM 3-MB-PP1) was not included in the western blots for *Spt5*-P, *Spt5*, *Rpb1,* or *tubulin*.

Supplementary Figure 11

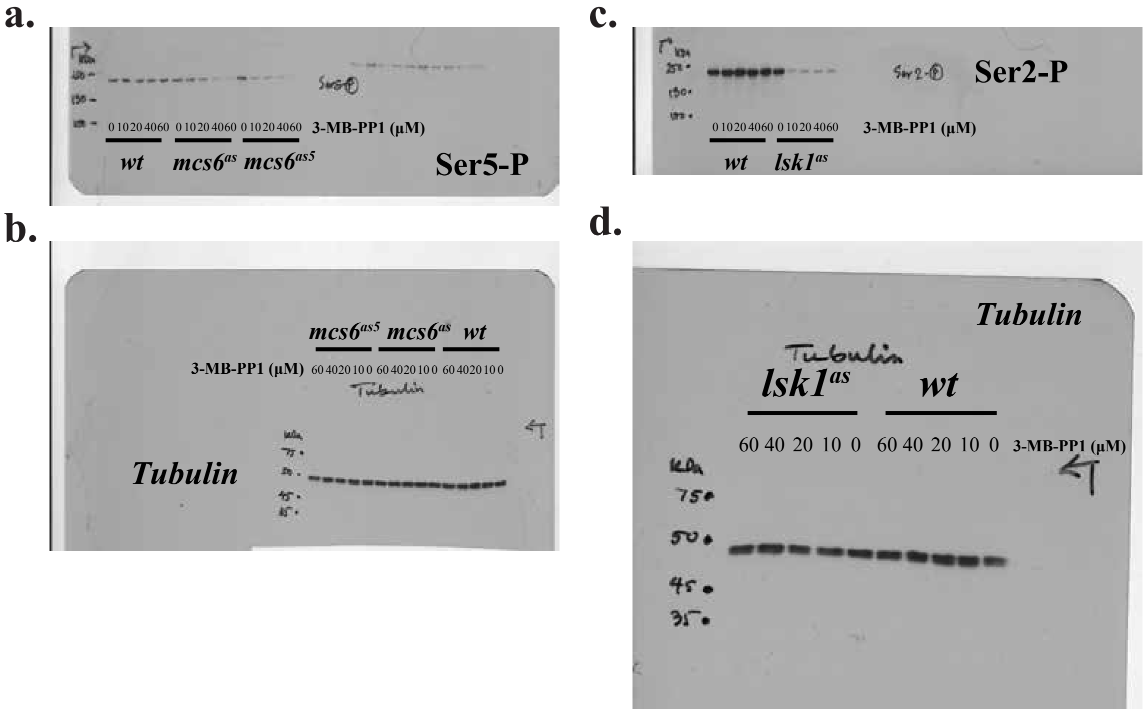

Supplementary Figure 11 | Western blot raw images. a & b. Raw scans of separate western blots used in Supplementary Figure 3b. Total protein extracts were prepared from *wt, mcs6^{as}* and *mcs6^{as5}* strains treated for 1 hour with the listed concentrations of 3-MB-PP1. **a.** Western blot using antibody against phosphorylated Ser5 residues within the CTD of *Rpb1*. **b.** Western blot using antibody against *tubulin*. **c & d.** Raw scans of separate western blots used in Supplementary Figure 3c. Total protein extracts were prepared from *wt,* and *lsk1^{as}* strains treated for 1 hour with the listed concentrations of 3-MB-PP1. **c.** Western blot using antibody against phosphorylated Ser2 residues within the CTD of *Rpb1*. **d.** Western blot using antibody against *tubulin*.

SUPPLEMENTARY REFERENCE

1. Love, M. I., Huber, W. & Anders, S. Moderated estimation of fold change and dispersion for RNA-seq data with DESeq2. *Genome Biol.* **15,** 550 (2014).
